# Fat mass prediction equations and reference ranges for Saudi Arabian Children aged 8–12 years using machine technique method

**DOI:** 10.7717/peerj.10734

**Published:** 2021-02-23

**Authors:** Rabab B. Alkutbe, Abdulrahman Alruban, Hmidan Alturki, Anas Sattar, Hazzaa Al-Hazzaa, Gail Rees

**Affiliations:** 1 School of Biomedical sciences, University of Plymouth, Plymouth, UK; 2 College of Computer and Information Sciences, Majmaah University, Majmaah, Saudi Arabia; 3 King Abdulaziz City for Science and Technology, Riyadh, Saudi Arabia; 4 Lifestyle and Health Research Center, Health Sciences Research Center, Princess Nourah Bint Abdulrahman University, Riyadh, Saudi Arabia

**Keywords:** Body fat, Children, Obesity, Machine learning, Predictive equation

## Abstract

**Background:**

The number of children with obesity has increased in Saudi Arabia, which is a significant public health concern. Early diagnosis of childhood obesity and screening of the prevalence is needed using a simple in situ method. This study aims to generate statistical equations to predict body fat percentage (BF%) for Saudi children by employing machine learning technology and to establish gender and age-specific body fat reference range.

**Methods:**

Data was combined from two cross-sectional studies conducted in Saudi Arabia for 1,292 boys and girls aged 8–12 years. Body fat was measured in both studies using bio-electrical impedance analysis devices. Height and weight were measured and body mass index was calculated and classified according to CDC 2,000 charts. A total of 603 girls and 374 boys were randomly selected for the learning phase, and 153 girls and 93 boys were employed in the validation set. Analyses of different machine learning methods showed that an accurate, sensitive model could be created. Two regression models were trained and fitted with the construction samples and validated. Gradient boosting algorithm was employed to achieve a better estimation and produce the equations, then the root means squared error (RMSE) equation was performed to decrease the error. Body fat reference ranges were derived for children aged 8–12 years.

**Results:**

For the gradient boosting models, the predicted fat percentage values were more aligned with the true value than those in regression models. Gradient boosting achieved better performance than the regression equation as it combined multiple simple models into a single composite model to take advantage of that weak classifier. The developed predictive model archived RMSE of 3.12 for girls and 2.48 boys. BF% and Fat mass index charts were presented in which cut-offs for 5th, 75th and 95th centiles are used to define ‘under-fat’, ‘normal’, ‘overfat’ and ‘subject with obesity’.

**Conclusion:**

Machine learning models could represent a significant advancement for investigators studying adiposity-related issues in children. These models and newly developed centile charts could be useful tools for the estimation and classification of BF%.

## Introduction

Obesity has now become a worldwide health issue in both adults and children. The percentage of people who live with obesity in Saudi Arabia, according to World Obesity Federation, is 57% of adults and 36.4% of children (World Obesity, 2020). Saudi Arabia and Kuwait have the highest rate of childhood obesity in the Middle East and North Africa (Farrag, Cheskin & Farag, 2017). Childhood is considered as a critical phase due to the growth and development of tissues and organs, and also the ability to influence future health and risk of disease by influencing the growth trajectory (Corvalan et al., 2007). Many researchers have investigated the prevalence of obesity in children, however the methods of measuring children and predicting obesity need to be adapted to the target population to fully address the situation.

Obesity is defined as an excessive fat mass in relation to the total body mass (World Health Organization, 2020). Body mass index (BMI (kg/m^2^)) percentile is a commonly used method to identify overweight, (between the 85th and 95th percentiles), and obesity (at the 95th percentile or greater for children and teenagers) (CDC, 2020). Raised BMI indicates a high body weight however it does not distinguish between body compartments (body fat, muscle mass and skeletal mass). Therefore, the wide use of this method could affect the estimation of obesity in different populations (Ballabriga & Carrascosa, 2006).

Another way to assess overweight or obesity is by measuring or predicting body fatness, expressed as percent body fat in children (Fortuño et al., 2003). Therefore, measuring the body fat percentage (BF%) for children is likely to be more accurate in assessing adiposity than calculating BMI (Freedman & Sherry, 2009). An increase in body fat is highly correlated with several chronic diseases in childhood such as diabetes and, could lead to morbidly in adulthood (Going et al., 2011; Dietz, 1998). The increase of the adipose tissue in children with obesity is associated with the accumulation of immunological cells such as macrophages. This could potentially lead to major inflammatory cytokine production that could stimulate adipose tissue inflammation and dysfunction (Weisberg et al., 2003). Obesity is also considered a key risk factor for numerous types of cancer in adulthood (Barberio et al., 2019).

Many studies have confirmed that in explaining the causes of metabolic syndrome the distribution of body fat is more important than merely excess adiposity (Shuster et al., 2012; Kwon, Kim & Kim, 2017; Lee et al., 2016). Although BMI is an adequate index of adiposity to describe populations, it has become clear that this simple anthropometric measurement has to be accompanied by other indices of body shape (such as the waist circumference or the waist-hip ratio) to identify patients with overweight/obesity who have a high-risk pattern of body fat. In recent decades the use of new forms of imaging have come into general use for such medical diagnoses (Louise Thomas et al., 2000; Graffy & Pickhardt, 2016).

The Dual-energy X-ray absorptiometry (DXA) is considered as the gold standard method to measure body composition (Scafoglieri & Clarys, 2018). This method provides separate readings for body composition such as fat percentage, lean mass, adipose and mineral content. Although DXA is valid and accurate, it should be undertaken by a trained medical technician or radiologist. DXA and other laboratory based methods are not applicable and available for routine clinical practice and in studies with large cohort sample sizes. In a young age, it would not be appropriate to use X-rays (even in small doses). Other high technical methods that can objectively measure fat in children such as hydrostatic weighing (HW); air displacement plethysmography (ADP), isotope dilution (total body water), and total body electrical conductivity (TOBEC), however, they are limited for clinical experience and very small numbers of participants.

Numerous researchers use bioelectrical impedance analysis (BIA) tools to estimate the BF% in children in large studies. BIA assesses differences in impedance caused by the fat and lean tissues when a very small current pass through the body. These tools are less invasive for children than other aformentioed methods and are portable, practical and have been validated against other methods (Lee et al., 2017). Although imaging (DEXA) and other techniques are available and accurate but are not suitable for routine clinical assessment of body fat. Simple methods (particularly weight, height) based on easy measurements and their validity would be of great value.

Other studies have generated equations to predict body fat and established reference ranges by using traditional statistical analysis (Cameron et al., 2004; Katch & McArdle, 1973). Machine learning algorithms make predictions with high performance, on the other hand, conventional statistical models aim at inferring relationships between independent and dependent variables. The benefits those advanced algorithms, such as gradient boosting, comprise flexibility and scalability compared with conventional statistical approaches, which makes it deployable for several tasks, such as diagnosis and classification (Rajula et al., 2020).

The simplicity in implementing these equations could help to avoid the high costs of data collection and the fragility of those models in the cases of missing variables. However, a new approach has been considered as a promising technology in diseases prediction, classification and promoting human health—machine learning (ML). Numerous studies have applied ML approaches to either predict or intervene in health problems (Dijkhuis et al., 2018). Using a machine learning algorithm for modelling such data to predict BF% enable predictive capacity, and ease of use in real world. The robustness for modelling a complex data, and especially, allow more systematic analysis of large cohorts with many predictor variables in contrast to traditional statistical methods. Such algorithm enables fitting large number of data along with large number of features/variables to be learned from. However, in this study we show that with 1,292 data points, the algorithm were able to achieve high performance and accuracy in comparison to traditional statistical methods such as linear regression.

Hammond et al. (2019) used ML in order to predict the obesity status at age five using data collected during the first two years. Hammond’s study found that ML can predict childhood obesity in the future. Therefore, there is an opportunity to use the ML approach to develop a prediction equation in Saudi Arabia as a screening tool to not only predict the body fat but also to establish reference ranges.

Classifying the BF% in children necessitates referring to a reference range with variable cutoff values for different ages. There is a lack of body fat reference ranges in children in the Middle East region. However, other nations such as USA and UK have established national body fat reference charts for children; nevertheless, they are of limited use in certain populations and may not be utilised worldwide (Laurson, Eisenmann & Welk, 2011; McCarthy et al., 2006).

Therefore, this paper has two objectives. The first is to develop statistical equations to predict BF% for Saudi children using simple parameters (age, sex, weight and height) by employing machine learning technology. The second objective is to establish a reference range for BF% in children aged 8–12 years in Saudi Arabia.

## Materials and Methods

### Subjects

Data were drawn from two different studies that used a cross sectional design and were conducted in schools in the two major cities in Saudi Arabia (the capital, Riyadh, and the holy city of Makkah). The two different studies used similar techniques and procedures. The data were collected from boys and girls in grades three (age 8–9 years), four (age 9–10 years), five (age 10–11 years) and six (age 11–12 years). In Riyadh, only subjects with a healthy weight and obesity participated as the aim of the Riyadh study was to compare data between children with a heathy weight and obesity only. While in Makkah only girls participated as the aim was focusing on girls with different body comopsitions.

The schools in Makkah were randomly chosen by the education department in each area to obtain a representative sample of the city’s schoolgirls (three governmental schools and four private schools). In Riyadh the researcher randomly selected the schools from five districts to represent the city. Two primary governmental schools (one for boys and one for girls) along with two private schools were randomly selected (one boys’ school and one girls’ school). Overall, a total of nineteen schools were selected to participate in the two studies. More details of the methodology regarding the inclusion and exclusion criteria, the response rate, and the sample size are stated for both studies have been documented in Alkutbe (2017) and Alturki, Brookes & Davies (2018).

### Anthropometric and body fat measurement

The measurements were conducted in the school laboratory as described in Al-Kutbe et al. (2017) (Makkah) and Alturki, Brookes & Davies (2018) (Riyadh). The data in the first study were collected over a period of 7 months in 2014, and the data for the second study were collected over a period of 4 months December 2015 to March 2016 (Al-Kutbe et al., 2017; Alturki, Brookes & Davies, 2018). In both studies, height was measured to the nearest 0.1 cm using a portable stadiometer (Seca, UK) and measurements were repeated until within 0.1 cm. The children were asked to stand in bare feet on the marks of the foot board and take off any upper hair clips. Their heads were adjusted to Frankfurt plane (the horizontal line from their ear canal to the lower border of the eye socket). The body weight and BF% were measured in the two studies by two different bioelectrical impedance tools. The Tanita Body Composition Analyser (TBF-300M/TBF300MA, Birmingham, UK) was used in Makkah and the Omron BF511 (model HBF-511B-E) was used in Riyadh. Different devices were used to measure BF%, however, both methods have similar measuring protocols and they are both validated to measure BF%. Both studies were conducted on Saudi children population and non-Saudi children were excluded. In both studies, body weight and BF% were measured by two different bioelectrical impedance tools according to manufacturer’s instruction for this population. Each participant was asked to stand bare feet on the device, and participant’s information (height, age and gender) were entered into the device for calculation and waited for body composition’s reading

The details concerning the basic technique and the aspects that can influence the anthropometric measurements in both studies have been documented in details in Al-Kutbe et al. (2017) and Alturki, Brookes & Davies (2018).

The participant was asked to stand with bare feet for a few seconds for the reading. The BMI was calculated as weight (kg)/height (m)^2^. The BMI was then plotted on the 2,000 CDC growth reference curve to classify the children with obesity (95> centile), overweight (86–94 centile); normal weight (5–85 centile) and underweight (<5 centile).

### Ethics approval and consent to participate

In the Makkah study, ethical approval was granted by the Faculty of Science and Technology Human Ethics Committee, University of Plymouth. The School Health Affairs Committee in Saudi Arabia (which has authority over projects conducted in schools) also assessed the risks and procedures involved in the study and approved the project. Following this, the Projects Management Committee in the General Directorate of School Education in Makkah granted permission for the involvement of the schools. Parents and children were fully informed about the study aims, requirements and use of their data, and both parents and child gave written informed consent. The Riyadh study was approved by the Institutional Review Board (IRB) at the Ministry of Health in Saudi Arabia (approval number 15-336E). The Ethical Clearance for Research Involving Human Participants was reviewed and obtained from The University of Queensland Behavioural and Social Sciences Ethical Review Committee (BSSERC) (approval number 2015001629). In addition, permission to conduct the study at primary schools was obtained from the Ministry of Education in Riyadh. Written informed consent was obtained from parents/guardians of the children who participated in the study.

### Statistical analysis

#### Modelling prediction equation

The current provided analysis covers the main contribution of the study this by analysing the most influencing features along with its weights, also covers the algorithms details and the exact parameters values in which this makes the experimentation reproducible to others using the same fitted data.

The analysis in this study only focuses on participants with healthy weight and obesity categories as underweight and overweight samples are excluded from the analysis for reasons mentioned in the method section. The entire original data set was (1,292 boys and girls aged 8–12 years) and a 69 participants were excluded due to missing data. In the girls’ dataset, 603 randomly selected samples were used for the learning phase models and 153 is the size of the validation set. In the same manner, for the boys’ dataset, 374 randomly selected samples were used for training the models and 93 was used as a validation set.

Many factors were tested to fit the best model; separate models were generated according to gender. The BMI was included as it improves the model even though weight and height have already been included. Therefore, weight, height, BMI and child’s age were the features set and used for the statistical analysis. Two regression models were trained and fitted with the construction samples and tested with the validation samples to estimate the continuous variable—fat percentage. The cross-validation (CV) approach was used to train and validate the base models. Using CV tends to generally decrease the probability of overfitting to the construction data set. The dataset was split randomly to ensure that the train and test subsets are representative of the original dataset. In which this prevents the learned algorithm from overfitting. The dataset was split into five consecutive folds with shuffling. Each fold was then used once as a validation set while the remaining four-folds formed the training set. Using a simple function, such as regression model, is always recommended as a starting point when conducting such experimental analysis. It is easy to interpret the developed model’s parameters and provide more understandable approach of what factors (measures) influence the dependent variable (fat parentage in this case). However, such simple models are not expected to fit very well as the simplicity sometimes comes with cost in which the performance of estimation could be not the best among other sophisticated algorithms

In order to achieve a better estimation result, gradient boosting algorithms has also been employed, which consist of a machine learning technique that can be used for regression and classification problems, that produces a prediction model in the form of an ensemble of weak prediction models, typically decision trees. It builds the model in a stage-wise fashion as other boosting methods do, and it generalizes them by allowing optimization of an arbitrary differentiable loss function. Gradient boosting implementation in scikit-learn—Python library has the variable importance which measures how significantly a given feature is biased towards correlated predictor variables as presented in the result section (Strobl et al., 2008). For measuring the loss, the root means squared error (RMSE) equation was used which measures the average of the squares of the errors—that is, the average squared difference between the estimated values by the trained algorithm and what is estimated. Equation (1) explains how the RMSE is computed as *x* denoted to the estimated vales and y denoted the actual value to be estimated.

Equation 1: Root Mean-Squared Error (RMSE).

(1) $${\rm RMSE}(X, Y) = \left[\frac{1}{n}\sum^{n}_{i=1} (x_i - y_i)^{2}\right]^{1/2}$$

In order to test if there was a significant difference between the ground truth fat percentage and the estimated fat percentage of the validation set, a *t*-test was used to compute the *p*-value between these two sets, the validation and the resulted estimated values. Sensitivity and specificity are statistical measures used to evaluate the performance of a binary classification test. Therefore, these metrics were not used for evaluation the developed model in this study as the research problem is predicting a regression value and not a binary class. Using more suitable measures such as RMSE provides more accurate results to evaluate such model.

#### Body fat reference range

The BF% obtained from both studies for those of a healthy weight and those with obesity were used to determine the reference range adjusted by age and gender. In order to establish a reference range for BF%, the 95% interval that could be estimated by assuming a normal distribution from the data was used (Kirkwood & Sterne, 2010). The general assumption that values within the 95 interval from the lower limit 2.5th to the upper limit 97.5th percentile is included in the range (Wayne, 2008).

Accordingly, the lower and upper limit of the 95% prediction interval were used to determine the healthy range reference since the healthy range cannot be determined from all groups to avoid skewing the upper and lower limit. The fat percentage from the group with obesity was used to classify the group between the healthy range and obesity where the lower limit for the group with obesity and the upper limit for the healthy weight determined the overfat range. The analyses were performed using the IBM Statistical Package for the Social Sciences, version 23 (SPSS Inc., Chicago, IL, USA).

## Results

After the exclusion a total number of participants (1,223 boys and girls aged 8-12 years) were divided into gender groups, 756 girls and 467 boys. Table 1 presents the BMI for the girls and boys datasets descriptive and comparative statistics.

**Table 1:** Dataset descriptive and comparative analysis for BMI for girls and boys datasets.

Gender	Category	No. of Samples	BMI µ	BMI σ	BMI Median	BMI Maximum	BMI Minimum
Girls	healthy weight	452	17.28	2.06	17.00	27.00	13.60
	Obese	304	26.47	2.85	26.30	36.07	18.00
Boys	healthy weight	236	17.43	2.04	17.20	28.00	13.71
	Obese	231	27.70	3.26	27.45	46.70	21.30

**Note:**

Abbreviations: BMI: body mass index; µ: arithmetic mean; σ: SD, BMI Kg/m^2^.

**Figure 1:**
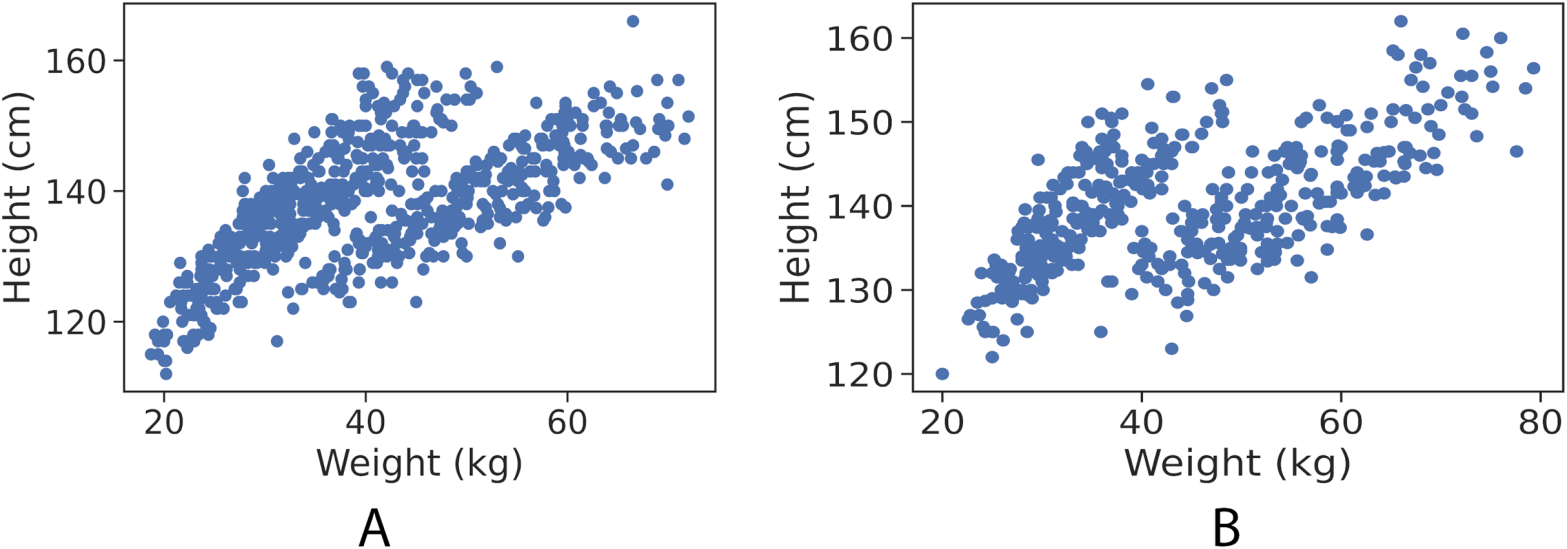
Data points (height in cm, weight in kg) for girls and boys. (A) Girls data points. (B) Boys data points.

By visualising each gender observation, as shown in Figs. 1A and 1B, it is apparent that the data points are clustered into almost two distinct patterns. Each of which corresponds to the category type that the data point belongs to (healthy weight and with obesity).

In the experimental analysis, each gender’s samples were tested separately, in which the fitted models are only learned from one class (gender type) at a time. As explained in the statistical analysis section, five-folds cross-validation approach was used and the model trained and learned from the whole data points (independent model for each gender), it almost has no bias to age, weight or gender. Table 2 shows the learned parameters of the regression equation.

**Table 2:** Regression model coefficients and bias parameters.

Gender	Model coefficients				Bias
	Age	Weight	Height	BMI	
Girls	−0.19	0.21	−0.12	1.18	−23.09
Boys	−1.018	−0.06	0.0409	1.57	−0.55

The gradient boosting regressor was tweaked by hyperparameter tuning, using a grid-search approach to find the best model parameters combination as follows: ‘learning_rate’: 0.3, ‘max_depth’: 5, ‘max_leaf_nodes’: 5, ‘n_estimators’: 15. Table 3 presents the prediction results for both the linear regression and gradient boosting regressor models in estimating the dependent variable—fat percentage.

**Table 3:** Fat percentage estimation results.

Variable	Algorithm	Gender	RMSE	Variance score	*p*-Value
Body fat (%)	LR	Girls	4.44	0.75	0.54
		Boys	4.53	0.76	0.70
	GBR	Girls	3.12	0.88	0.99
		Boys	2.48	0.93	0.99

**Note:**

Abbreviations: RMSE: Root mean squared error; LR: linear regression; GBR: a gradient boosting regressor.

The results presented in Table 3 shows that there is no significant difference between the control and validation groups as the *p*-values in all conducted tests are not significant.

**Figure 2:**
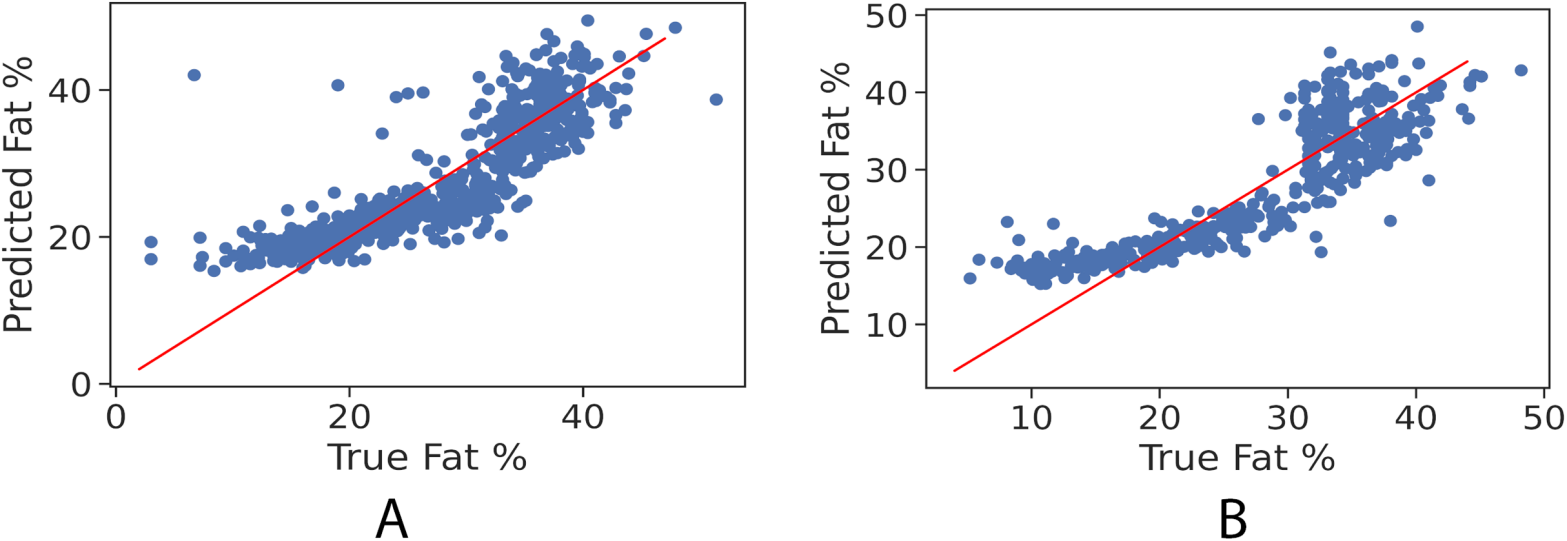
True vs. predicted fat % of the regression model for the girls’ and boys’ control set. The red trendline represents the fitted regression line learned by the trained model. (A) Girls’ control set; (B) Boys’ control set.

**Figure 3:**
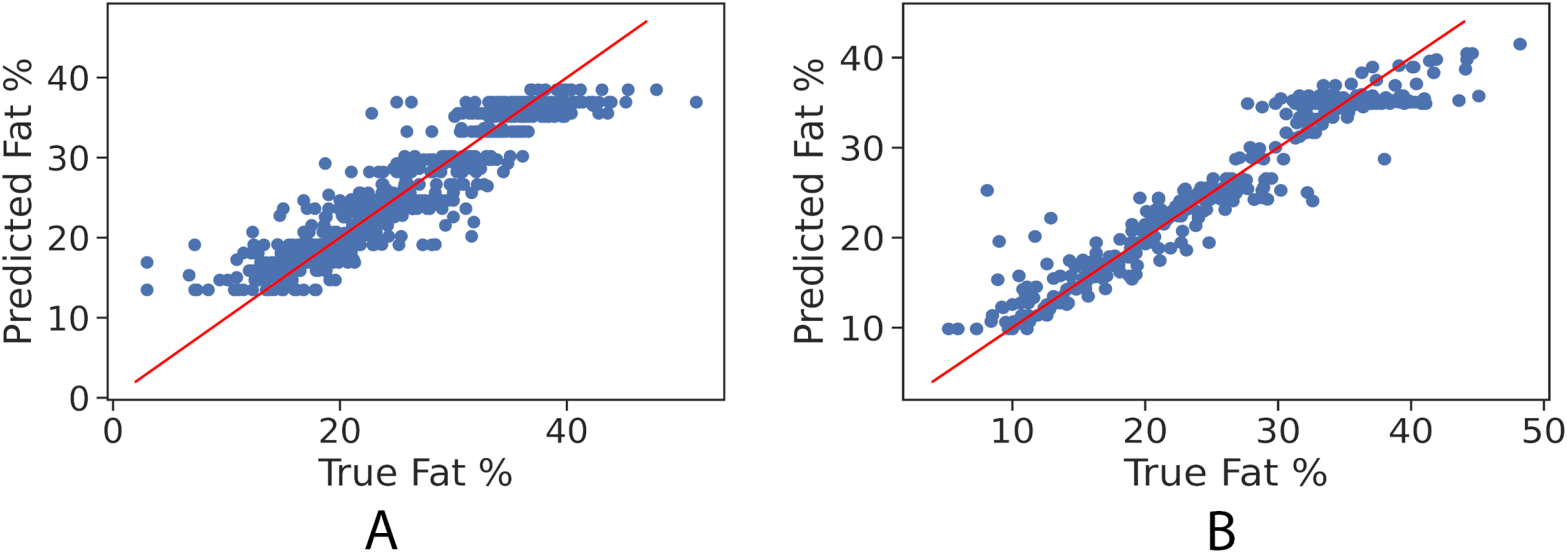
True vs. predicted fat % of gradient boosting regression model for the girls’ and boys’ control set. Red line represents the fitted regression line learned by the trained model. (A) Girls’ control set; (B) Boys’ control set.

In order to further examine the goodness-of-fit of the trained models, Figs. 2A and 2B illustrate the true fat percentage along with the estimated value by the regression models of the girls and boys sets respectively. It is apparent that a large number of the estimated fat percentage deviate from the true values. This is due to using a simple regression model for both categories, the healthy weight and with obesity. It is expected by using individual models for each data group will significantly improve the prediction performance, since the variance within one group is less than the variance when having two groups. For the gradient boosting models, it can be seen in Figs. 3A and 3B that the predicted fat percentage values are more aligned with the true value than those in regression models. It is not surprising that the gradient boosting achieved better performance as combines multiple simple models into a single composite model to take advantage of that weak classifier.

**Figure 4:**
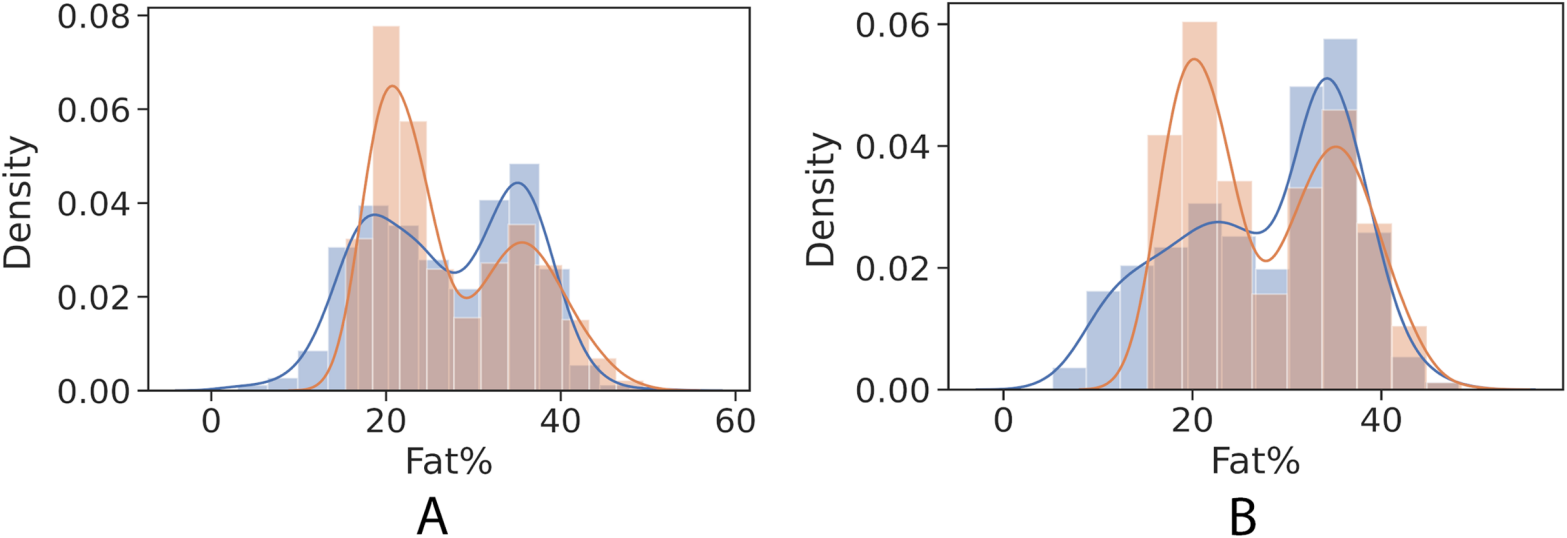
Control univariate observations of regression model. The blue curve represents the true values of fat percentage. The green curve represents the predicted values of fat percentage by the trained model distribution. (A) Girls; (B) Boys.

**Figure 5:**
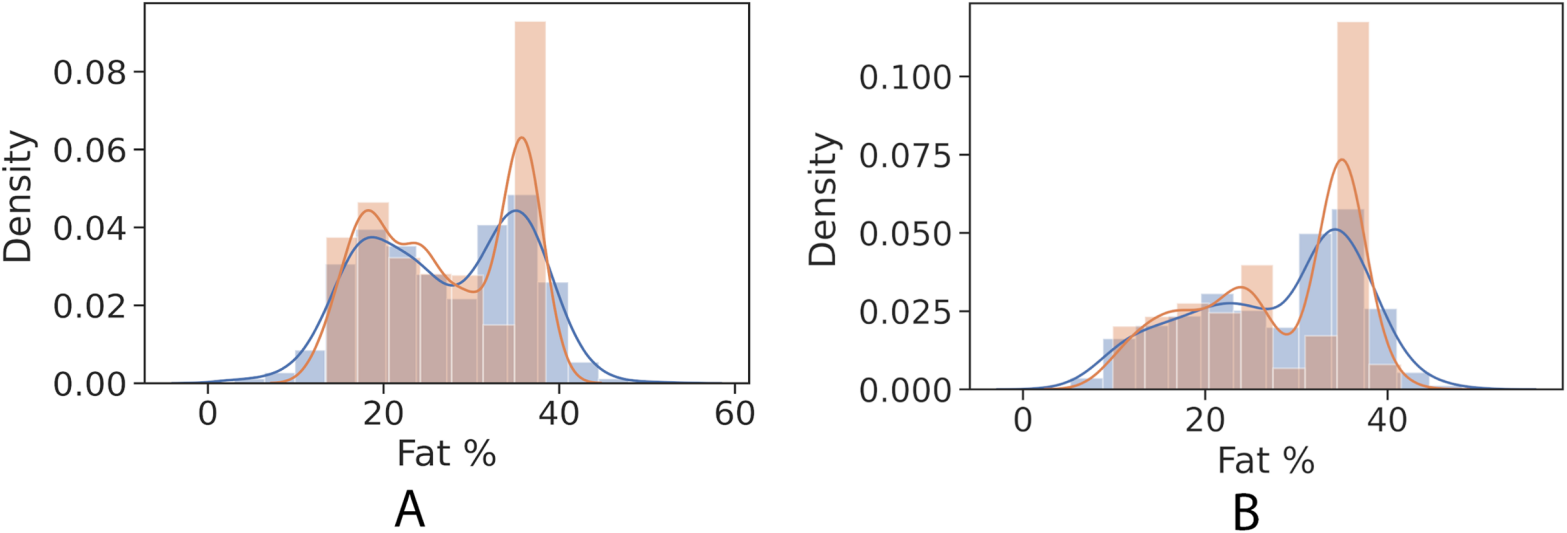
Control univariate observations distribution of gradient boosting regression model. The blue curve represents the true values of fat percentage. The green curve represents the predicted values of fat percentage by the trained model distribution. (A) Girls; (B) Boys.

Figures 4 and 5 show kernel density estimate and histogram, which indicate that the gradient boosting has better density estimation in comparison with the regression model.

**Figure 6:**
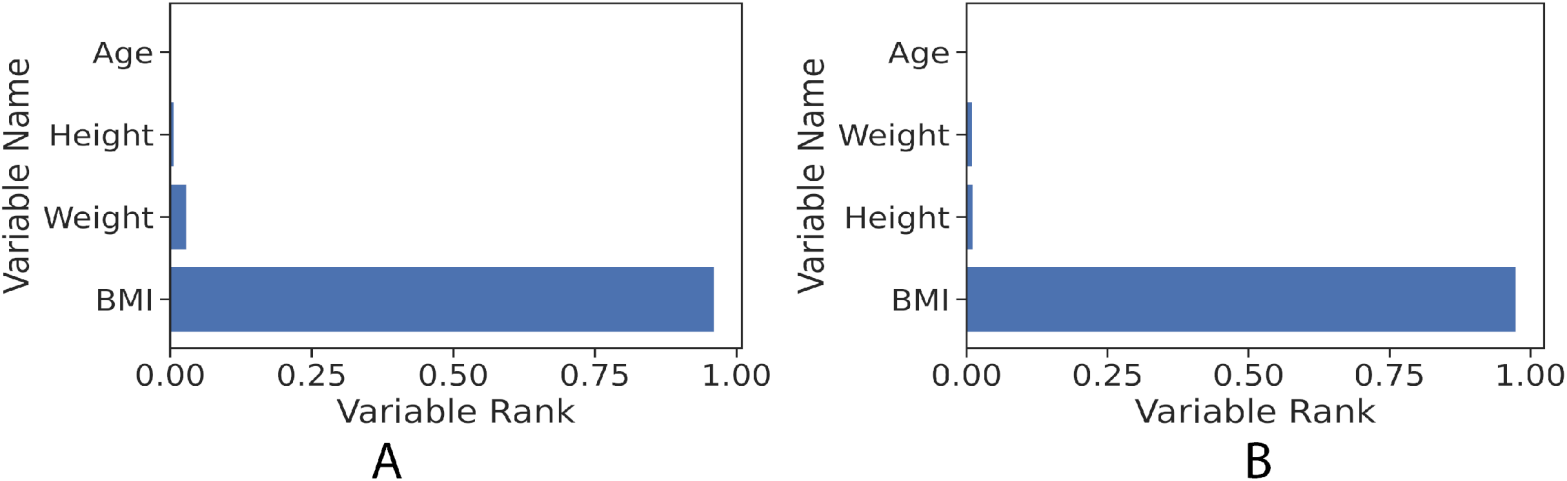
Feature rank of gradient boosting regression model for the girls and boys sets. The figure shows clearly that BMI value in both girls and boys models has the most influence power to the learned models towards predicting the fat percentage. (A) Girls; (B) Boys.

The gradient boosting implementation in the scikit-learn library has the feature of measuring how significant each independent variable is towards estimating the target value (fat percentage in the context of this study). Figure 6 illustrates the features’ ranks and it can be seen that the highest rank is BMI. The resulted measurement ranks are: BMI: 0.96, weight: 0.015, height: 0.02 and age: 0.0005.

The preliminary analysis of the BF% for children in this study illustrates that set of BF% reference range for the boys and girls. The tabulated data are listed in Table 4. The healthy weight girls aged 9–10 years show a similar range to the boys up to age of 10 years but are then strikingly different in body fat % range. The healthy reference range in girls from age 10 continues to diverge slightly from the boys.

**Table 4:** The classification levels of body fat percentage (% fat) based 95% interval.

Gender	Age (years)	Body fat percentage classifications			
		Lean	Healthy	Overfat	Obese
Boys	9–10	≤9.4	9.5–25.0	25.1–28.5	≥28.6
	10–11	≤9.7	9.8–27.8	27.9–31.9	≥32.0
	11–12	≤9.3	9.4–27.0	27.1–30.9	≥31.0
Girls	8–9	≤13.5	13.6–21.7	21.8–23.5	≥23.6
	9–10	≤9.7	9.8–27.5	27.6–31.5	≥31.6
	10–11	≤11.6	11.7–27.4	27.5–31.0	≥31.1
	11–12	≤12.8	12.9–28.5	28.6–32.0	≥32.1

Table 5 shows the total number of participants who were classified as healthy weight and with obesity according to BMI in the previous studies (Al-Kutbe et al., 2017; Alturki, Brookes & Davies, 2018) and were classified according to BF% by using our equations. Noticeably, the total number in the healthy weight group according to BMI is more than the total number of healthy weights according to their BF% in the boys. Similarly, in girls, the total number of healthy weights according to their BF% is 18% less than the total number of healthy weights according to BMI. Also 299 girls were classified as having obesity according to their BF% which is less than the number of subjects with obesity according their BMI.

**Table 5:** The classification of participants according to body fat percentage and BMI, and mean of body fat percentage using both methods (direct measurements, and predicted equation).

Classified according		Boys		Girls	
		HW	OB	HW	OB
Bf % predicted	Number of participants	214	237	372	306
	Bf % predicted	19.44 ± 4.81	35.40 ± 2.31	20.46 ± 3.96	35.83 ± 1.83
	Bf % measured	19.48 ± 5.68	35.37 ±3.50	20.15 ± 5.37	36.20 ± 3.66
BMI	Number of participants	236	231	452	304
	Bf % predicted	20.055 ± 6.38	35.47 ± 2.26	21.49 ± 4.75	35.70 ± 2.06
	Bf % measured	20.054 ± 5.59	35.47 ± 3.42	21.21 ± 5.95	36.11 ± 3.71

**Note:**

Abbreviations: HW healthy weight; OB obesity.

There was no significant difference between the BF% using the equation and by bioelectrical impedance measurement of BF% in all groups. Table 6 shows that fat mass index (FMI) references adjusted by sex and age.

**Table 6:** Fat mass index percentile reference data for boys and girls.

Percentiles								
	Age (years)	5	10	25	50	75	90	95
Boys	9–10	1.57	2.05	3.33	5.81	9.24	10.14	11.18
	10–11	1.60	2.17	3.68	5.69	9.86	10.98	12.49
	11–12	1.78	2.27	3.45	8.48	10.12	11.13	12.26
Girls	8–9	1.92	2.10	2.54	3.22	6.40	9.48	10.42
	9–10	1.97	2.34	3.18	5.38	8.82	10.34	11.18
	10–11	1.96	2.34	3.15	5.19	8.90	10.49	12.01
	11–12	2.36	2.67	3.85	6.12	9.49	11.13	11.85

## Discussion

In this study, a novel predictive equation was developed to estimate BF% in children aged 8–12 years in Saudi Arabia using machine learning. This equation was derived from 1,292 children of both genders. This equation was developed using easily obtained parameters - weight, height, age and sex of children. It was found that the gradient boosting algorithm performed better than the simpler linear regression model. The developed predictive model achieved a RMSE of 3.12 for girls and 2.48 for boys. This indicates that the developed model (GBR) is able to predict boys’ fat percentage with less error than the girls test set.

Recently, increasing number of studies reporting BF% using multiple anthropometrics measurements (Aristizabal, Estrada-Restrepo & Giraldo García, 2018; Ferenci & Kovács, 2018; Hastuti et al., 2018; Henry et al., 2018). Using machine-learning equations to measure BBF% could significantly Save time and money. In this study, we report a highly accurate BF% calculation equation based on very minimum anthropometric measurements and significantly higher participants number in comparison to other similarly developed models (Henry et al., 2018, Uçar et al., 2021).

The main strengths of this study are the convenience of using our equation as a screening tool for obesity based on BF% in comparison to actual measurement of BF which is not always at the disposal of health care personnel and the equipment needed can be expensive. The advantages of using the prediction model are that it is a non- invasive tool in comparison to preforming skinfold measurements and easy to use in comparison to methods such as BIA, and highly accurate in estimate the BF%.

Most other validation studies used a method of directly measuring body fat to validate their equation, however, in our novel study, this was a limiting factor. Nevertheless, in this study total body fat results from a bio-impedance body composition analyser were used to estimate the BF% and to develop the equation, similarly, the bio-impedance tool was used in a national UK study to create children sex-specific centile curves for body fat (McCarthy et al., 2006).

Bio-impendence has been validated in other studies (Kabiri, Hernandez & Mitchell, 2015) against the gold standard dual-energy X-ray absorptiometry (DEXA) in children and found moderately strong absolute agreement correlation. Both the Tanita and Omron BIA analysers have been validated against other methods. Tanita showed an excellent test-retest reliability and was used in our study to develop the predicted BF% formula. Moreover, the Omron tool was assessed for its reliability against DEXA in children and adolescents and showed a reliable result in predicting BF% (Mooney et al., 2011; Talma et al., 2013). Moreover, our sample size is large in comparison to other studies and statistically normally distributed which gives a strength to this study.

When the BF% is used to classify the children; it gives more accurate figures of body fat than using BMI. Accordingly, the number of girls in the healthy weight group using BF% was 372, which was 17.8 % less than healthy weight according to BMI. Similarly, the number of boys in the healthy weight group according to BF% was 214, which was approximately 9% less than healthy weight according to BMI. Consequently, using BMI lead to the misclassification of 80 girls and 22 boys. Therefore, using BMI could cause misclassification of body status in children, which potentially could lead to inappropriate implementation of interventions.

Interestingly, the misclassification in girls is more than double in comparison to boys, and this could be explained by the fact that some girls reach puberty earlier than their peers. At puberty girls undergo physiological changes, and this will lead to changes in body composition and especially increases in body fat.

The age at which girls reach puberty varies based on several factors, including race and country of origin. In Saudi Arabia, a study by Al-Agha, Saeedi & Tatwany (2015) showed that females reach B2 tanner stage, which is defined as the presence of breast buds, at nine years old. These might explain our findings that showed girls aged 9–10 have higher BF% (Alnwsany et al., 2015).

Therefore, this paper aimed to generate the FMI reference range to improve the assessment of body composition over the use of BMI. As BMI cannot distinguish between fat mass and fat-free mass, whereas; FM is a component of FMI formula. Using FMI is a more justifiable measure to estimate body composition in children and thus not affected by the variability of the fat-free mass.

The BF% of participants with a healthy BMI were analysed for normal distribution and results higher and lower limits were excluded and a reference range for BF was established. This reference number was then compared to all participants and showed that contradictable to what was observed, some healthy weight children classified were unhealthy based on our reference range and vice versa.

A possible limitation is that only subjects with healthy weight and obesity were included in this study because one of the studies that generated a portion of this data excluded the overweight and underweight. However, overweight subjects are not required to establish the reference range for BF% since establishing such reference range requires healthy participants only (Kirkwood & Sterne, 2010). This was determined by the range of healthy weight and with obesity.

A second limitation of our study that we were unable to account for biological maturity changes, which could be perceived in the body size of participants of the same chronological age, because our data were obtained from two different studies neither of them consider these biological differences. Moreover, this study was not taking into account the pubertal status of participants which could have an effect on the predictive measurements and should definitely be considered in future work.

Another limitation is that we were unable to validate our equation or formula with the gold standard method (DXA) or other possible techniques (e.g. Magnetic resonance imaging (MRI)), however, our novel method was generated using Gradient boosting which is considered as the most robust technique for establishing predictive models (Lipton et al., 2015). Our Gradient boosting models will also be available for use and could help in partitioning of weight into its components which could be valuable for monitoring children health.

In conclusion, a novel equation was created and validated that could be used for simple screening/diagnostics, treatment and follow-up of obesity based on simple parameters. In addition, a new reference range for BF% was established for children aged 8–12 years in Saudi Arabia.

## Conclusions

Our predicting models and charts could be used as a screening tool for children in schools for early diagnosis and implementation of interventions to avoid future obesity-related complications. A future study should validate our novel predictive formula against the gold standard method to further confirm accuracy. Moreover, a future study using a larger sample size and a wider age range is preferable to cover the full childhood range of BF%. These prediction tools and reference charts could be used to aid the assessment of BF% in children and allow the early implementation of lifestyle advice.

## Supplemental Information

10.7717/peerj.10734/supp-1 Supplemental Information 1 Raw data.

10.7717/peerj.10734/supp-2 Supplemental Information 2 Codebook for raw data.

## Additional Information and Declarations

### Data Availability

The following information was supplied regarding data availability: The raw measurements and codebook are available in the Supplemental Files.
